# Encapsulation and Enhanced Release of Resveratrol from Mesoporous Silica Nanoparticles for Melanoma Therapy

**DOI:** 10.3390/ma14061382

**Published:** 2021-03-12

**Authors:** Diogo Marinheiro, Bárbara J. M. L. Ferreira, Párástu Oskoei, Helena Oliveira, Ana L. Daniel-da-Silva

**Affiliations:** 1 Department of Chemistry & CICECO-Aveiro Institute of Materials, University of Aveiro, 3810-193 Aveiro, Portugal; diogomarinheiro@ua.pt; 2 Department of Biology & CESAM-Centre for Environmental and Marine Studies, University of Aveiro, 3810-193 Aveiro, Portugal; parastu.oskoei@ua.pt (P.O.); holiveira@ua.pt (H.O.)

**Keywords:** melanoma, resveratrol, mesoporous silica nanoparticles, drug delivery

## Abstract

Chemotherapy has limited success in the treatment of malignant melanoma due to fast development of drug resistance and the low bioavailability of chemotherapeutic drugs. Resveratrol (RES) is a natural polyphenol with recognized preventive and therapeutic anti-cancer properties. However, poor RES solubility hampers its bioactivity, thus creating a demand for suitable drug delivery systems to improve it. This work aimed to assess the potential of RES-loaded mesoporous silica nanoparticles (MSNs) for human melanoma treatment. RES was efficiently loaded (efficiency > 93%) onto spheroidal (size~60 nm) MSNs. The encapsulation promoted the amorphization of RES and enhanced the release in vitro compared to non-encapsulated RES. The RES release was pH-dependent and markedly faster at pH 5.2 (acid environment in some tumorous tissues) than at pH 7.4 in both encapsulated and bulk forms. The RES release from loaded MSNs was gradual with time, without a burst effect, and well-described by the Weibull model. In vitro cytotoxicity studies on human A375 and MNT-1 melanoma cellular cultures showed a decrease in the cell viability with increasing concentration of RES-loaded MSNs, indicating the potent action of the released RES in both cell lines. The amelanotic cell line A375 was more sensitive to RES concentration than the melanotic MNT-1 cells.

## 1. Introduction

Malignant melanoma is the deadliest form of skin cancer and one of the most challenging malignancies to treat, with a steeply rising incidence and poor prognosis in advanced stages [1]. In recent decades, the prevalence of melanoma has been continuously increasing worldwide [2,3], and despite representing only 1% of skin cancers, it results in a large majority of skin cancer-related deaths [4]. Chemotherapy is an important instrument in the treatment of metastatic melanoma and in the palliation of patients with resistance to immunotherapies [5,6,7]. However, single-agent chemotherapy has seen limited success in melanoma treatment due to the fast development of drug resistance, poor targeting efficiency, and low bioavailability of most chemotherapeutic drugs [1,6].

Resveratrol (3,5,4′-trihydroxystilbene; RES) is a naturally occurring polyphenolic phytoalexin. It can be found in the skin and seeds of more than 70 different plant species, including many foods commonly consumed in the human diet such as grapes, berries, grains, tea, and peanuts [8]. RES is found in nature as both cis and trans isomeric forms, with the latter being more abundant and more biologically active [9]. When resveratrol is exposed to light, trans-RES conversion to cis-RES occurs via photoisomerization [10]. The pharmacological research interest in trans-RES emerged after Siemann and Creasy [11] reported its presence in red wines, and some epidemiological studies, such as the one of Renaud and Lorgeril [12], simultaneously correlated red wine consumption to the low incidence of cardiovascular diseases in the French population, a phenomenon known as the “French paradox”. The reduced risk of cardiovascular disease in this population, despite the high intake of saturated fats, has been associated with red wine’s high consumption. Nowadays, RES is well-known for its antioxidant, anti-inflammatory, cardio protective, and neuroprotective properties [13]. Additionally, it has shown potential in the treatment of various disorders mediated by free radicals and oxidative stress [14,15], as well as metabolic disorders [16], and it has also demonstrated marked preventive and therapeutic anti-cancer activity [17,18]. Specifically, resveratrol can reverse multidrug resistance in cancer cells, and, when used in combination with clinically used drugs, it can sensitize cancer cells to standard chemotherapeutic agents [19,20].

Despite its tremendous therapeutic potential, RES suffers from several pharmacokinetic limitations that hinder its pharmaceutical and clinical applications. Namely, RES exhibits a short biological half-life, chemical instability (due to its tendency to suffer oxidation and extreme photosensitivity), extensive and rapid metabolism and elimination, low aqueous solubility, and poor bioavailability [21]. To overcome these drawbacks, the use of nanostructured carriers has emerged and demonstrated potential to enhance the solubility, chemical stability, bioavailability, and biological activities of RES [22,23,24]. Examples of nanoplatforms that have been proposed include liposomes [25,26,27,28], cyclodextrins [29,30,31], solid lipid nanoparticles [32,33,34], polymeric micelles [35], and polymeric nanoparticles [36,37,38]. However, these carriers suffer from limitations related to poor stability, low drug loading, the use of organic solvents, and high production costs [39]. In contrast, mesoporous silica nanoparticles (MSNs) exhibit several superior features in comparison to other nanocarriers such as a high drug loading capability due to their high surface area and pore volume, tunable mesopore size and pore/shape connectivity, easy surface functionalization, controllable degradability in biological environments, high in vitro and in vivo biocompatibility, and a high level of clearance and excretion [40]. Hence, in the last years, there was a growing interest in using MSNs as nanocarriers for the encapsulation and delivery of RES [41,42]. Recent studies have reported the therapeutic efficacy of RES-loaded MSNs against colon [43], gastric [44], and prostate [45] cancer. The apoptosis in human melanoma cells induced by RES has been previously reported [46,47]. However, the effect of encapsulated RES on the treatment of melanoma remains largely unexplored and limited to polymer-based nanocarriers [37].

This work aimed to prepare RES-loaded MSNs and investigate their therapeutic potential in human melanoma. An important parameter that dictates the bioavailability and hence the biological effect is drug release. Thus, the in vitro release of RES from MSNs was evaluated and the kinetic data were analyzed through several release models. Next, the effect of RES-loaded MSNs on cell viability of human A375 and MNT-1 melanoma cellular cultures was investigated.

## 2. Materials and Methods

### 2.1. Chemicals

All chemicals were reagent grade and used without further purification unless otherwise specified. Hexadecyltrimethylammonium bromide (CTAB; 99%), tetraethyl orthosilicate (TEOS; 99%), phosphate-buffered saline (PBS) pH 7.4 (0.01 M; with NaCl 138 mM and KCl 2.7 mM), and dialysis cellulose tubing membrane (3.5 kDa cut off) were purchased from Sigma-Aldrich (St. Louis, MO, USA). Triethanolamine (TEA; 99%) and sodium chloride (NaCl; 99.5%) were obtained from Fisher Chemical (Loughborough, UK). Trans-resveratrol (99%) was purchased from Tokyo Chemical Industry (TCI Europe N.V., Zwijndrecht, Belgium). Methanol (CH_3_OH; >99%) and ethanol (CH_3_CH_2_OH; >99%) were acquired from VWR International (Radnor, PA, USA). All chemicals were used as received without further purification. Deionized water was used in all experiments and was produced using a Milli-Q system with a 0.22 μm filter (Synergy Equipment, Millipore, Burlington, MA, EUA).

### 2.2. Synthesis of MSNs

The MSNs were prepared by a simple method adapted from the literature [48] and based on a (an aqueous) biphasic system. Briefly, 2.0 g of surfactant (CTAB) were dissolved in 20 mL of Milli-Q water. The solution was then moved to a round-bottom flask immersed in an oil bath under constant stirring (450 rpm). Then, when the solution reached 95 °C, 100 µL of TEA were added, and the resulting solution was stirred for one hour. Afterwards, 1.5 mL of TEOS was added dropwise, and the reaction was left to occur for 6 h. The temperature was controlled during all synthesis processes, and the reaction occurred in reflux conditions to ensure the maintenance of concentrations. MSNs were collected by centrifugation (13,000 rpm for 10 min) and washed three times with ethanol to remove the residual reactants. All the collected materials were extracted for 4 h with sodium chloride solution (NaCl; 1 wt.%) in methanol at room temperature to remove the CTAB template. One last wash with ethanol was made to remove the residual NaCl. Finally, for the complete removal of surfactant, MSNs were calcinated in a muffle furnace (Termolab) for 10 h at 550 °C.

### 2.3. Loading of RES

Two distinct methods were used for loading the RES into the MSNs: the rotary evaporation and immersion techniques.

#### 2.3.1. Rotary Evaporation Technique

RES loading was performed using the rotary evaporation technique [2,3]. Briefly, 40 or 80 mg of RES were dissolved in 10 mL of ethanol and sonicated for 4 min. Afterwards, 100 mg of the MSN were added to the solution, and the dispersion was placed in the ultrasonic bath for 10 min. The solvent was then slowly evaporated using a rotary evaporator at 50 °C, until all ethanol was removed, to obtain the RES-loaded MSNs. The samples are designated hereafter by MSN/RES30 and MSN/RES40 based on the nominal weight percentage of RES. The final solids were scratched off from the round-bottom flask, stored at room temperature, and protected from light with aluminum foil.

#### 2.3.2. Immersion Technique

The adopted procedure was identical to the one used in the rotary evaporator method; however, after suspending the MSNs in the RES solution, the resultant suspension was stirred at room temperature for 65 h while protected from light. The final solids were collected by centrifugation (13,300 rpm for 10 min) and stored while protected from light with aluminum foil.

### 2.4. Physicochemical Characterization of the Materials

FTIR spectra were collected on a Mattson 7000 spectrometer (Mattson Instruments Inc, Madison, USA) using the KBr technique; 256 scans were collected per sample over the range of 4000–300 cm^−1^ at a resolution of 2.0 cm^−1^. The size and morphology of MSNs were investigated by scanning and transmission electronic microscopy (STEM) using a Hitachi H-9000 instrument (Hitachi, Tokyo, Japan) operating at 300 kV. Samples were prepared by evaporating dilute suspensions of the nanoparticles in ethanol on a copper grid coated with an amorphous carbon film. X-ray diffractograms were recorded using a PANalytical Empyrean diffractometer (Malvern PANalytical, Malvern, UK) with Kα(Cu) radiation and a curved graphite monochromator (step 0.05°) in the range 10° < 2θ < 65°. Zeta potential measurements were performed with a Zetasizer Nano Plus instruments (Malvern Instruments, Malvern, UK) by suspending the nanoparticles in deionized water at different pH values (2, 4, 6, 8, and 10).The established equilibration time was 120 s (for each sample), and 3 measurements were performed with 100 runs each. Nitrogen adsorption−desorption isotherms at 77 K were measured on a Micrometrics Gemini V2.0 (Micromeritics Instrument Corp., Norcross, GA, USA) system. All samples were pre-treated for 5 h at 473 K under nitrogen before measurements. The pore size was calculated from desorption branches of isotherms by the Barrrett–Joyner–Halenda (BJH) method. Surface areas were calculated by the Brunauer–Emmett–Teller (BET) method. TGA was employed to access the amount of RES loaded on the MSNs with a Hitachi STA300 instrument (Hitachi, Tokyo, Japan) at a heating rate of 5 °C/min, from 30° to 800 °C, under a nitrogen:oxygen atmosphere (3:1). The elemental analysis of carbon and hydrogen was performed on a Leco TruSpec-Micro CHNS 630-200-200 (LECO, Saint Joseph, MI, USA) was. Heating curves of the materials were obtained using a differential scanning calorimeter (Diamond DSC, Perkin Elmer, Waltham, USA). Accurately weighed samples (2–5 mg) were loaded into a non-hermetically crimped aluminum pan and heated under a nitrogen purge at the rate 50 mL/min, from 30 to 350 °C at a heating rate of 10 °C/min. Data were analyzed using TA Instruments’ Universal Analysis 2000 software V4.5A. UV–VIS spectra were obtained in a GBC Cintra 303 spectrophotometer (GBC Scientific Equipment, Victoria, Australia) operating in the range of 200–500 nm.

### 2.5. In Vitro Release Studies

Release studies were performed in PBS at two different pH values of 7.4 and 5.2. The suspensions used in the release studies were prepared by dispersing RES-loaded MSNs (equivalent to 500 µg of bulk RES) into 1 mL of PBS. The suspension was introduced into a dialysis membrane (3.5 kDa cut-off) and immediately immersed in 29 mL of PBS. The solution was kept under continuous but gentle stirring at 37 °C while protected from light. The concentration of released RES was monitored for 72 h. After a specific period of time, aliquots of solution (1 mL) were withdrawn for analysis and immediately replaced with 1 mL of PBS. For comparison, the test was also performed using bulk RES.

The aliquots were analyzed to measure their absorbance with an UV–Vis spectrophotometer (GBC, Cintra 303). The concentration of resveratrol in the aliquots was calculated based on the Lambert–Beer law, which correlates the absorbance with the concentration of the sample. In order to perform this analysis, calibration curves were created for the resveratrol solutions at pH 7.4 and 5.2. For fixed, known concentrations of resveratrol, the absorbance at λ = 305 nm was measured and plotted against the concentration, as shown in Figures S1 and S2 (Supplementary Materials) with a good linear correlation (R^2^ = 0.9997 and R^2^ = 0.9992 for pH 7.4 and 5.2, respectively). The absence of a peak or strong absorption in the spectral region of 260–287 nm indicated that there was no formation of cis-RES or degradation compounds during the RES release studies (Figure S3, Supplementary Materials) [49].

The cumulative release percentage of RES was calculated using Equation (1),

(1) $$\mathrm{Cumulative}\;\mathrm{release}\;\left(\%\right)=\frac{\mathrm{Volume}\;\mathrm{of}\;\mathrm{aliquot}\;\mathrm{withdrawn}\;\left(\mathrm{mL}\right)}{\mathrm{Total}\;\mathrm{volume}\;\left(\mathrm{mL}\right)}\times P_{\left(t-1\right)}+P_{t}$$

where P_t_ corresponds to the percentage released at time t and P_(t−1)_ corresponds the percentage released previous to time t. The data presented in the release curves are an average of triplicates.

### 2.6. Cell Studies

#### 2.6.1. Reagents

Dimethyl sulfoxide (DMSO; ≥99.7%) and 3-(4,5 dimethyl-2-thiazolyl)-2,5-diphenyl tetrazolium bromide (MTT; 98%) were purchased from Sigma-Aldrich (St. Louis, MO, USA). The MNT-1 cell line was kindly provided by Dr. Manuela Gaspar (iMed.ULisboa, Portugal), and the A375 cell line was purchased from the European Collection of Authenticated Cell Cultures (ECACC) and supplied by Sigma-Aldrich (Madrid, Spain). Dulbecco’s Modified Eagle’s Medium, fetal bovine serum (FBS), l-glutamine, and fungizone (250 U mL^−1^) were from Gibco, Life Technologies (Grand Island, NY, USA), and penicillin–streptomycin (10,000 U mL^−1^) was from Grisp (Porto, Portugal). Trypsin– ethylendiaminetetraacetic acid EDTA (0.25% trypsin and 1 mM EDTA) was purchased from Gibco, Life Technologies (Grand Island, NY, USA).

#### 2.6.2. Cell Culture

Both cell lines, MNT-1 and A375, were aseptically grown in Dulbecco’s Modified Eagle’s Medium, supplemented with 10% (FBS, 2 mM l-glutamine, 1% penicillin–streptomycin (10,000 U mL^−1^), and 1% fungizone (250 U mL^−1^) at 37 °C in a humidified atmosphere with 5% CO_2_. Cells were observed daily for confluence and morphology using an inverted phase-contrast Eclipse TS100 microscope (Nikon, Tokyo, Japan). Sub confluent cells were trypsinized with trypsin–EDTA (0.25% trypsin and 1 mM EDTA) when monolayers reached 70% confluence.

#### 2.6.3. In Vitro Cell Viability Assays

Cell viability was determined by the colorimetric MTT assay, which measures the formation of purple formazan in viable cells [50]. MNT-1 cells were seeded in 96-well plates at 3500, 2000, and 1500 cells well^−1^ for 24, 48, and 72 h of exposure, respectively, while A375 were seeded at 3500, 2500, and 2000 cells well^−1^ for 24, 48, and 72 h of exposure, respectively. After 24 h, medium was replaced with fresh medium containing: (i) pristine MSNs (0, 25, 50, 100, 200, and 400 µg mL^−1^), (ii) bulk RES (0, 50, 100, 200, 400, and 700 µM), or (iii) RES-loaded MSNs (0, 25, 50, 100, 200, and 400 µg mL^−1^). Cells exposed to the control medium were used as a negative control, and cell viability was measured after 24, 48, and 72 h. At the end of the incubation time, the wells were emptied and washed with PBS to remove remaining particles, and then fresh medium (100 µL) was placed in each well. After that, 50 μL of MTT (1 g L^−1^ in PBS) was added to each well and incubated for 4 h at 37 °C in a 5% CO_2_ humidified atmosphere. Thereafter, the culture medium with MTT was removed and replaced by 150 μL of DMSO for formazan crystal solubilization. The samples absorbance (Abs) was measured with a BioTek Synergy HT plate reader (Synergy HT Multi-Mode, BioTeK, Winooski, VT, USA) at 570 nm.

## 3. Results and Discussion

### 3.1. Preparation of MSNs

**Figure 1:**
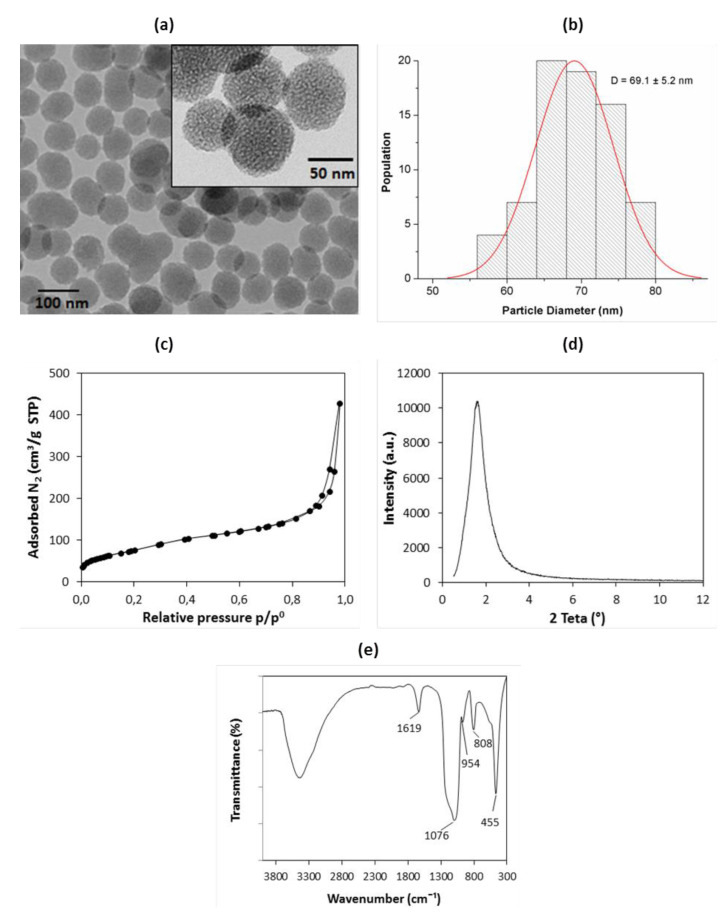
Physicochemical characterization of mesoporous silica nanoparticles (MSNs). (**a**) TEM micrographs (inset—high magnification); (**b**) histogram of particle size distribution; (**c**) N_2_ adsorption–desorption isotherms at 77 K; (**d**) low angle XRD diffraction pattern; (**e**) FTIR spectrum with main bands identified.

The as-synthesized samples were characterized by TEM, and the results are shown in Figure 1a,b. The MSNs showed a spheroidal morphology with an average particle size of 69.1 ± 5.2 nm, a uniform size distribution, and a porous structure (Figure 1a inset). The N_2_ adsorption–desorption isotherm (77 K) was type IV with a hysteresis loop (Figure 1c), which is characteristic of mesoporous materials [51]. The MSNs presented a hysteresis loop at a high relative pressure, above 0.8, that may be attributed to inter-particle porosity [52,53]. The specific surface area was 266 m^2^/g, with an average pore size of 9.4 nm. The surface area was relatively low compared to other MSNs prepared using NaOH as a catalyst [54]. These results were similar to some of the values found when employing organic amine catalysts such as triethanolamine (TEA) [45,55,56]. TEA was used because it generally acts as a growth inhibitor for MSNs and allows for the better control of particle size to values lower than 100 nm, which is desirable for drug delivery applications. The XRD pattern (Figure 1d) only consisted of a broad peak at small angles (1.6°) that suggested a low ordering porous structure [57]. The FTIR spectrum (Figure 1e) displayed bands centered at 1076, 808, and 455 cm^−1^ that were ascribed to the asymmetric Si–O–Si stretching, symmetric Si–O–Si stretching, and O–Si–O bending modes of SiO_2_, respectively. The bands at 1619 cm^−1^ indicated water molecules adsorbed onto siliceous materials, while the band at 954 cm^−1^ were ascribed to silanol (Si-OH) groups. The absence of bands of the CTAB indicated that the surfactant was successfully removed. The elemental analysis detected only trace amounts of carbon (0.04 wt.%), which further confirmed that CTAB was totally eliminated.

### 3.2. Loading of RES

In a preliminary stage of the work, two different loading methods were used: the immersion method and the evaporation method. The initial amount of MSN and RES available for loading was identical in both approaches. With the aim of assessing the presence of RES in the loaded MSNs, FTIR spectra of the materials were acquired (Figure S4—Supplementary Materials. The spectrum of the sample loaded by immersion was similar to the spectrum of pristine MSNs. Conversely, several new bands were observed in the spectrum of the MSN loaded by evaporation. The new bands could be assigned to RES and were attributed to the O-H stretching of phenolic hydroxyl bonds (broad band at 3192 cm^−1^), benzene skeleton vibrations (bands from 1587 to 1445 cm^−1^), and the bending vibrations of C=C-H (987 and 966 cm^−1^) [58]. The results clearly indicated that the evaporation method was more efficient for loading resveratrol. This conclusion was further supported by XRD data (Figure S5). The XRD pattern of the particles loaded by immersion was very similar to the one of pristine MSNs. In contrast, the diffractogram of the particles loaded by evaporation showed sharp peaks that were ascribed to the crystalline form of RES. Though the initial RES concentration in the loading solution was identical in both approaches, the RES concentration increased as the solvent was evaporated in the evaporation method. This generated a driving concentration gradient that drove RES into the pores of MSNs and explains why the evaporation method was more efficient. Achieving higher loading efficiencies using the immersion method would require higher RES concentration in the loading solution, as was observed with other drugs [59]. However, this could be economically less effective.

**Figure 2:**
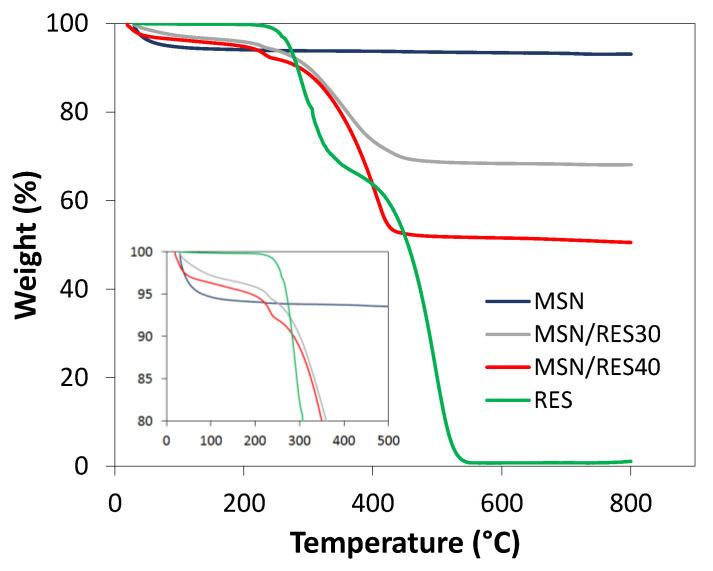
TGA curves of pristine MSN, resveratrol (RES), and loaded MSN/RES30 and MSN/RES40 samples.

The MSNs (100 mg) were loaded in the rotary evaporator using initial amounts of RES of 40 and 80 mg to yield the MSN/RES30 and MSN/RES40 samples, respectively. TGA was employed to determine the RES content in the loaded samples, i.e., the loading capacity. Both loaded samples exhibited a marked weight loss at temperatures above 200 °C that was ascribed to the thermal degradation of RES since the pristine MSNs showed an almost negligible weight loss (<0.9%) at those temperatures (Figure 2). The weight loss contribution of pristine MSNs was removed when computing the amount of RES in loaded samples. The results of loading efficiency (Equation (2)) and capacity are shown in Table 1. The loading capacities determined by TGA was 26.8% and 43.7% for the MSN/RES30 and MSN/RES40 samples, respectively. These values were in agreement with the carbon content of the loaded samples, as assessed by elemental analysis (Table S1). The expected and the measured drug loading were very close, and the loading efficiency was consequently high (nearly 94% and 98% for the MSN/RES30 and MSN/RES40 samples, respectively).

(2) $$\mathrm{Loading}\;\mathrm{efficiency}\;\left(\%\right)=\frac{m_{\mathrm{loaded}\;\mathrm{RES}}}{m_{\mathrm{initial}\;\mathrm{RES}}}\times 100$$

### 3.3. Characterization of RES-Loaded MSNs

**Figure 3:**
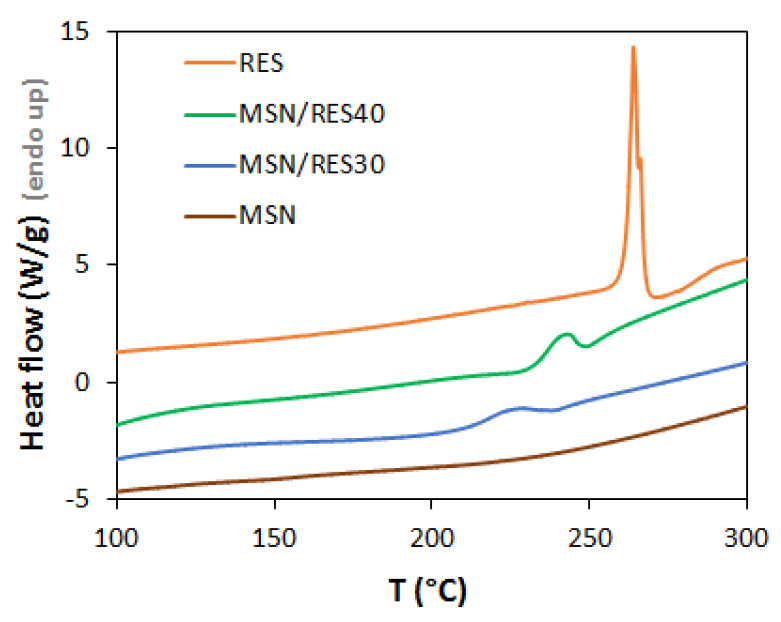
Differential scanning calorimetry (DSC) profile of pristine MSN, RES, and loaded MSN/RES30 and MSN/RES40 samples.

Loaded samples were further characterized by DSC and XRD, aiming to understand whether RES was in the crystalline or amorphous state since this influences RES solubility and release. During the loading process by evaporation, RES molecules can be accommodated inside the pores or adsorbed at the surface of MSNs. The confinement of RES in mesopores may result in the amorphization of the drug, a phenomenon that is affected by the pore size and surface chemistry of porous platforms [43,60]. Furthermore, some RES molecules may remain free, i.e., with no interaction with MSNs. The DSC curve of bulk RES (Figure 3) presented a sharp endothermic peak at 265 °C that is attributed to the melting transition of crystalline RES. No bulk-like melting transition was observed in the loaded samples, suggesting that there was no crystallization of RES outside the MSNs. Because DSC only detects crystalline RES, we cannot exclude the presence of non-encapsulated amorphous RES. Solvent endothermic peaks were not present, thus showing the successful removal of organic solvents in all samples. In the loaded samples (MSN/RES), smaller and broader endothermic peaks were found at lower temperature than the melting of bulk RES, suggesting that part of the RES confined on the pores underwent crystallization. Similar DSC observations were found for ibuprofen confined inside mesoporous silicon [61]. The lower melting temperature was ascribed to crystal size, which is restricted by pore dimensions inside the pores. The shift of the melting temperature can be correlated with the nanoconfined crystallite diameter according to the Gibbs–Thompson equation [62]. Thus, the reduction of the melting temperature was higher in MSN/RES30, indicating the formation of smaller crystallites than in MSN/RES40. The crystallinity degree of the loaded samples was estimated from the measured melting enthalpy, assuming the melting enthalpy inside the pores to be identical to the value of the bulk RES. The MSN/RES40 sample was slightly more crystalline than the MSN/RES30 sample (19.4% and 15.3%, respectively; Table S2—Supplementary Materials).

Overall, the DSC results indicated that the encapsulation led to the amorphization of part of the loaded RES. Most likely, the pore size of MSNs synthesized here (average size of 9.4 nm) was large enough to enable RES crystallization to occur to some extent. Previous studies employing MSNs with a smaller pore size (7 nm) reported the absence of the crystallization of encapsulated RES [43].

**Figure 4:**
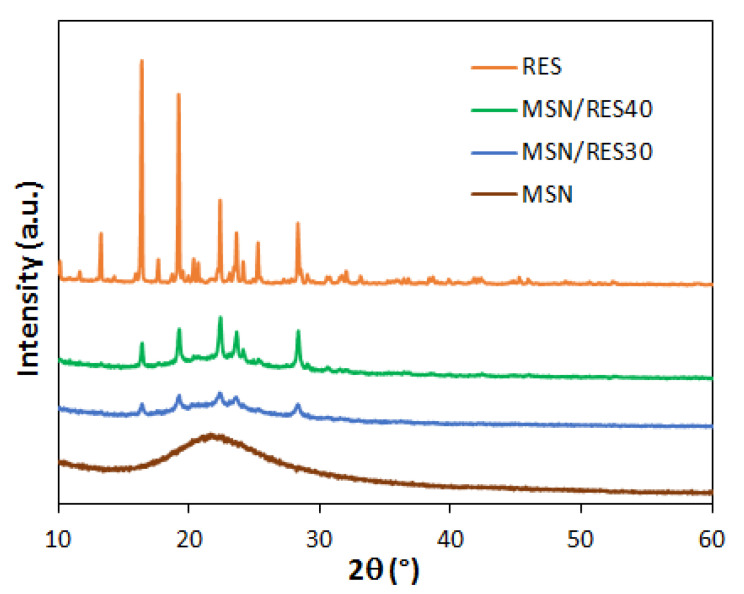
Wide angle XRD pattern of bulk RES, pristine MSNs, and RES-loaded MSNs.

The XRD results (Figure 4) were in line with DSC observations. The powder XRD of bulk RES exhibited multiple sharp diffraction peaks between 10° and 30° due to the crystalline state of the bulk RES. The intensity of the peaks dropped significantly in the diffractograms of the MSN/RES samples, confirming that the loading of RES on the MSNs promoted the amorphization of the drug. Overall, among the loaded samples, the RES reflections were more visible and intense in the MSN/RES40 diffractogram. This was in agreement with the higher crystallinity degree of MSN/RES40 determined by DSC. The intensity of the broad peak of the MSNs decreased after loading, a phenomenon that may be explained by the scattering effect of RES filling the mesopores [63].

**Figure 5:**
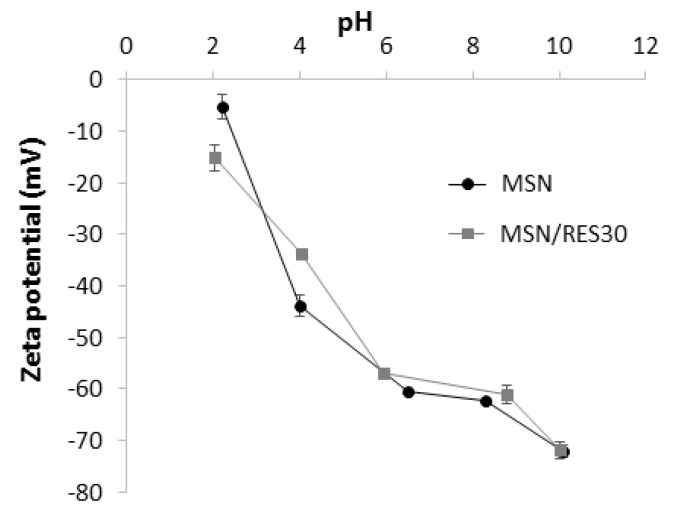
Zeta potential of pristine MSNs and RES-loaded MSNs at variable pH values.

Finally, zeta potential measurements were carried out to assess the surface charge of the RES-loaded MSNs at different pH values. Both pristine and loaded particles displayed a negative surface charge within the tested pH range (Figure 5). The zeta potential of loaded nanoparticles ranged from −15.2 mV (pH = 2) to −71.9 mV (pH = 10). Similar values were obtained for the pristine MSN, indicating that the RES loading did not markedly affect the zeta potential values of MSN. The negative zeta potential of the pristine MSNs was ascribed to the ionization of silanol groups on the surface of silica (vicinal, geminal, or isolated silanol) with distinct pKa values [64]. The silica crystallinity and pore arrangement might have also influenced the silica surface charge [65,66]. The marked pH dependence of the MSNs’ zeta potential observed here was in line with the results reported for vitreous (amorphous) silica and low ordering porous silica materials [65,66]. Particles with a high zeta potential magnitude exhibit an increased colloidal stability due to interparticle electrostatic repulsions. Typically, zeta potential values out of the range from +30 mV to −30 mV indicate colloidal stability [67], which suggests that at pH ≥ 4.0, it is possible to obtain stable suspensions for loaded and unloaded MSNs. However it should be stressed that our measurements were performed in deionized water. The presence of proteins and other biomolecules in a real physiological medium might affect the colloidal stability of nanoparticles due to the formation of a biomolecule corona [68].

### 3.4. In Vitro RES Release Studies

**Figure 6:**
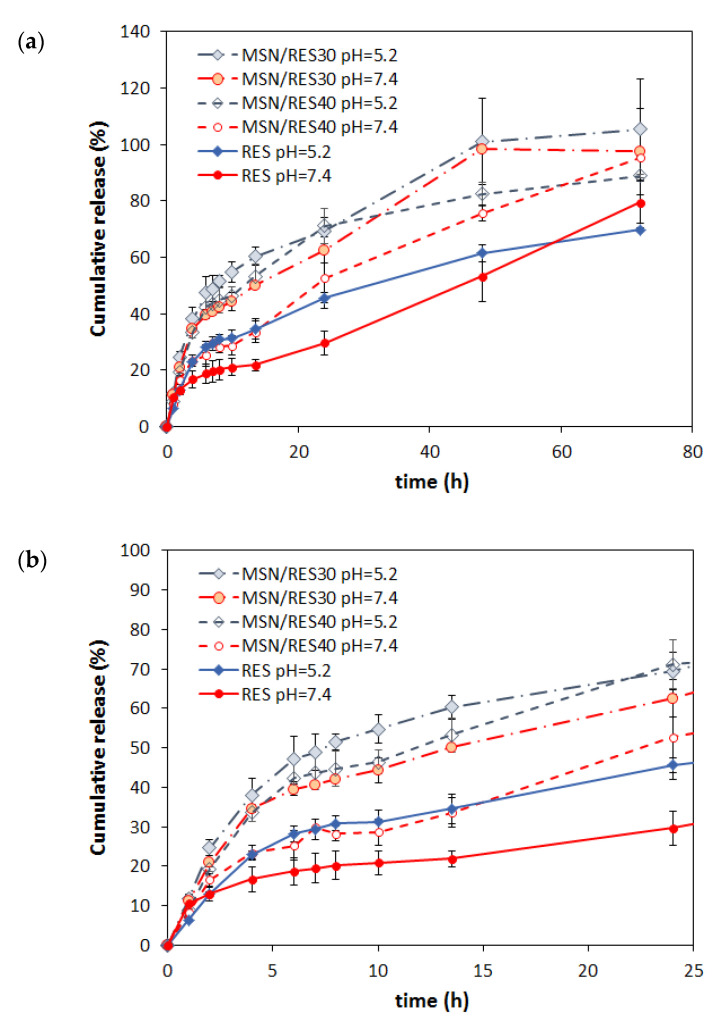
Cumulative release of resveratrol from the non-encapsulated (RES) and encapsulated samples (MSN/RES30 and MSN/RES40) at pH 7.4 and 5.2. (**a**) Full profile for 72 h and (**b**) the first 24 h.

Figure 6 shows the cumulative release of RES from the loaded MSNs at pH 7.4 (physiological pH) and 5.2 (acid environment in some tumorous tissues) in buffered solutions. For comparison, the release of bulk RES (non-encapsulated) was also evaluated. The RES release was pH-dependent, being markedly faster in acidic conditions in both encapsulated and bulk form. Thus, after 24 h, the RES release was 29.6%, 52.7%, and 62.5% at pH 7.4 for bulk RES and the MSN/RES40 and MSN/RES30 samples, respectively, and increased to 45.6 %, 71.1%, and 70.5%, respectively, at pH 5.2. The pH dependence of RES release could be ascribed to an increase of RES solubility when the pH decreases. The increased solubility of RES for pH values below 7.4 was reported in previous studies [49]. Interestingly, for longer release periods (72 h), the effect of pH on release is meaningless, and the cumulative released RES was identical at pH 7.4 and 5.2. Importantly, the RES-loaded MSNs provided an increased release of RES when compared with bulk RES at both pH values tested. The release of RES from loaded MSNs was gradual with time, without any burst effect. Only the MSN/RES30 sample, which contained less loaded RES, attained the equilibrium (plateau) within the tested 72 h period. Furthermore, the same sample attained the highest percentage of released RES, namely 97.5% and 105% after 72 h at pH values of 7.4 and 5.2, respectively.

In order to investigate the kinetics of the release of RES from MSNs and to gain further insight into the mechanism of release, three well-known models were applied to the experimental data: the Weibull model, the Korsmeyer–Peppas model, and an exponential equation based on the Noyes–Whitney equation and applying Fick’s law [69].

The Weibull model [69,70] is an empirical model described by Equation (3):

(3) $$M_{t}/M_{\infty }=1-e^{\left(-\frac{{\left(t-T_{i}\right)}^{\beta }}{\alpha }\right)}$$

where *M_t_* and *M_∞_* are the mass of the drug released at time *t*, *α* is the time process, *Ti* is the lag time before the onset of the drug release (in most cases zero), and *β* is the shape parameter that characterizes the curve as exponential (*β* = 1), S-shaped with upward curve followed by turning point (*β* > 1), or parabolic with higher initial slope after that consistent with the exponential (*β* < 1).

The Korsmeyer–Peppas (KP) model is a semi-empirical model based on Fick’s second law of diffusion for short time diffusion, as given by a power law equation (Equation (4)):

(4) $$M_{t}/M_{\infty }=k_{KP}\cdot \;t^{n}$$

where *M_t_*/*M_∞_* is a released fraction of drug at time *t* and *k_KP_* is a constant incorporating characteristics of the system [71]. The exponent *n* is indicative of the transport mechanism and also depends of the geometry of the system. In the case of spherical matrix, *n* = 0.43 corresponds to Fickian diffusion, 0.43 < *n* < 0.85 corresponds to non-Fickian diffusion, and *n* ≥ 0.85 corresponds to relaxation-controlled transport [72,73]. The model is valid for *M_t_*/*M_∞_* values up to 0.6.

The drug release can be interpreted as an exponential type of diffusion based on Noyes–Whitney and Fick’s law (NWF) (Equation (5)):

(5) $$M_{t}/M_{\infty }=1-e^{-k_{F}t}$$

where *M_t_* and *M_∞_* are the mass of RES released at time *t* and the total mass of RES loaded in the sample, respectively, and *k_F_* is the first-order rate constant independent of drug concentration that includes information on solvent accessibility to the substrate and the diffusion coefficient through the mesoporous channels [74,75].

The experimental data were fitted using non-linear regression by employing the method of least squares and the tool solver of the Excel software. The goodness of the fitting was evaluated based on the analysis of the statistical parameter coefficient of determination (R^2^) and chi-square (χ^2^).

The Weibull model better described the release kinetics data than the NWF equation (higher R^2^ and lower χ^2^ values), with a coefficient of determination (R^2^) in the range of 0.960–0.990 (Table 2). The Weibull model is a general empirical equation that has been successfully applied to several drug release systems, including to RES release from lipid and polymer-based carriers [76,77,78,79]. Because this is an empiric model, not deducted from any kinetic fundament, the model does not provide information about the mechanism of release [69]. In turn, the NWF equation is deducted employing the Fick’s first law of diffusion [75]. This equation has been successfully employed to describe the release of several drugs from non-swellable solid carriers, such as mesoporous silicas [75,80,81]. However, based on the analysis of the R^2^ and χ^2^ values (R^2^ ranged from 0.950 to 0.970), this model was less suitable to describe the experimental data than the Weibull model. Nevertheless, the differences observed on the release rate of the several samples could be correlated with the rate constant k_F_ given by the NWF equation fitting. Hence, a comparison of the k_F_ values showed that the RES release rate was higher for the MSN/RES30 sample and increased when the pH decreased from 7.4 to 5.2. The Korsmeyer–Peppas equation was adjusted to the experimental results for an RES release fraction of up to 60%, presenting correlation coefficients ranging from 0.964 to 0.975. For all the cases, the release exponent was in the range of 0.43 < *n* < 0.85, which corresponded to non-Fickian transport [69,73].

### 3.5. Cell Viability in Melanoma Cells

**Figure 7:**
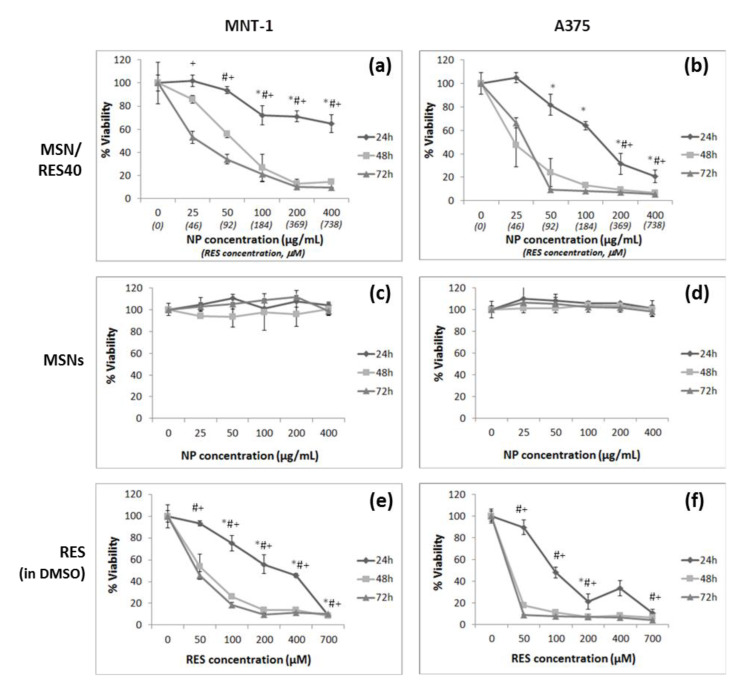
Effect of pristine MSNs, RES-loaded MSNs (0–700 μg/mL), and bulk RES (0–700 μM) on cell viability of MNT-1 and A375 melanoma cells by the 3-(4,5 dimethyl-2-thiazolyl)-2,5-diphenyl tetrazolium bromide (MTT) assay at 24, 48, and 72 h. Results are expressed as mean ± SD. * indicates significant differences between control at *p* < 0.05 for 24 h; # indicates significant differences between control at *p* < 0.05 for 48 h; and + indicates significant differences between control at *p* < 0.05 for 72 h. (**a**) MNT-1 cells exposed to RES-loaded MSNs; (**b**) A375 cells exposed to RES-loaded MSNs; (**c**) MNT-1 cells exposed to pristine MSNs; (**d**) A375 cells exposed to pristine MSNs; (**e**) MNT-1 cells exposed to bulk RES and (**f**) A375 cells exposed to bulk RES.

The cytotoxicity of the RES-loaded MSNs (MSN/RES40), bulk RES (non-encapsulated), and pristine MSNs in MNT-1 and A375 melanoma cells were evaluated using the MTT cell viability assay (Figure 7 and Table 3). The pristine MSNs did not affect cell viability, which was consistent with previous works regarding spherical MSNs interacting with melanoma cells [82]. Bulk RES (pre-dissolved in DMSO) reduced the cell viability of both cell lines, with the amelanotic cell line A375 being more sensitive to the RES concentration than melanotic MNT-1 cells. The RES concentrations that inhibited growth by 50% in MNT-1 cells were 253, 49.8, and 37.9 μM at 24, 48, and 72 h, respectively. In contrast, the concentrations that inhibited A375 growth by 50% were 113.4, 1.5, and 0.0026 μM at 24, 48, and 72 h, respectively. Notably, an RES concentration of 50 μM reduced the A375 cell viability to 8.6% after 72 h of exposure. While the MNT-1 cell line was tested herein for the first time with resveratrol, to our knowledge, the results of A375 were consistent with previous studies that reported the sensitivity of these cells to RES [46]. The RES-loaded MSNs (MSN/RES40 formulation) at 21.1 and 29.5 μg/mL significantly reduced the cell viability by 50% in A375 and MNT-1 cells after 48 and 72 h of exposure, respectively. An MSN/RES40 concentration of 131 μg/mL inhibited A375 cell growth by 50% after 24 h of exposure. In contrast, in the MNT-1 cells, the half maximal inhibitory concentration (IC50) at 24 h was not achieved for the range of the tested MSN/RES40 concentrations. The results suggested that A375 cells are more sensitive to RES-loaded MSNs than MNT-1 cells and agree with our observations when using non-encapsulated RES.

## 4. Conclusions

The polyphenol RES was successfully encapsulated onto MSNs with a high efficiency loading (93%) using the rotary evaporation method. The encapsulation promoted the amorphization of RES, which is desirable to increase the solubility of this drug. Through DSC and XRD analysis, signs of crystalline resveratrol were also found, suggesting that some RES might have crystallized within the MSNs pores. Future work should include reducing the size of the pores to prevent the crystallization of the RES without impairing the release. In vitro release assays revealed faster and enhanced RES release from encapsulated RES than from the bulk form due to RES amorphization. A pH-dependence of RES release was observed, being markedly faster at pH 5.2 than at pH 7.4. This pH-sensitive behavior is of high interest and may benefit RES delivery to tumor cells because the intracellular pH in tumor cells is lower than in healthy cells [83]. RES-loaded MSNs showed cytotoxicity in two melanoma cancer lines. The amelanotic cell line A375 was more sensitive to RES concentration than the melanotic MNT-1 cells. In summary, the results of our research indicate that RES-loaded MSNs are bionanoconjugates with the potential to treat melanoma. Other in vitro and in vivo studies are in progress to confirm the effectiveness of RES-loaded MSNs.

## Figures and Tables

**Table 1:** Loading efficiency and loading capacity of samples loaded using the evaporation method.

Sample	Loading Capacity (%)	Loading Efficiency (%)
MSN/RES30	26.8	93.7
MSN/RES40	43.7	98.1

**Table 2:** Kinetics parameters and goodness of the fits of RES release from loaded MSNs. k_F_: first-order rate constant independent of drug concentration; k_KP_: constant incorporating characteristics of the system; NFW: Noyes–Whitney and Fick’s law equation; K–P: Korsmeyer–Peppas model.

Model		MSN/RES30		MSN/RES40	
		pH 5.2	pH 7.4	pH 5.2	pH 7.4
NWF	**k_F_** (min^−1^)	1.41 × 10^−3^	1.05 × 10^−3^	1.12 × 10^−3^	6.0 × 10^−4^
	**R^2^**	0.9500	0.9530	0.9703	0.9694
	χ^2^	0.166	0.262	0.173	0.335
K–P	**k_KP_** (min^−n^)	0.0247	0.0256	0.01877	0.01343
	**n**	0.4865	0.4513	0.5090	0.4972
	**R^2^**	0.9751	0.9712	0.9636	0.9666
	χ^2^	0.033	0.028	0.043	0.024
Weibull	**α** (min^−β^)	121.3	164.6	87.12	429.0
	**β**	0.7214	0.7318	0.6357	0.8072
	**R^2^**	0.9749	0.9690	0.9902	0.9708
	χ^2^	0.044	0.055	0.034	0.097

**Table 3:** Estimation of half maximal inhibitory concentration (IC50) of bulk RES and RES-loaded MSNs to MNT-1 and A375 melanoma cells.

		24 h	48 h	72 h
**MNT-1**	**MSN/RES40** (μg/mL)	653.3	49.9	25.5
	**RES** (μM)	253.0	49.8	37.9
A375	**MSN/RES40** (μg/mL)	131.0	21.1	29.5
	**RES** (μM)	113.4	1.5	0.0026

## Data Availability

All data reported in this paper is contained within the manuscript and supplementary material.

## Supplementary Materials

The following are available online at https://www.mdpi.com/1996-1944/14/6/1382/s1. Figure S1: Calibration curve for RES quantification in PBS pH 7.4. Figure S2: Calibration curve for RES quantification in PBS pH 5.2. Figure S3: UV–Vis spectra (240–380 nm) of aliquots extracted along time during RES release studies of the loaded MSN/RES30 sample and non-encapsulated RES (pH 5.2). Figure S4: FTIR spectra of MSNs, RES, and loaded MSNs using the immersion (MSN/RES-ims) and evaporation (MSN/RES-evp) methods. The amount of MSNs (100 mg) and RES (40 mg) available for loading was identical in both methods. Figure S5: Wide angle XRD of pristine MSNs and loaded MSNs using the immersion (MSN/RES-ims) and evaporation (MSN/RES-evp) methods. Table S1: Elemental analysis of MSNs and loaded MSN/RES30 and MSN/RES40 samples. Table S2: Melting temperature and crystallinity degree of the samples.
