# Supplementary material for: Encapsulation and Enhanced Release of Resveratrol from Mesoporous Silica Nanoparticles for Melanoma Therapy
Source: Materials (Basel). 2021 Mar 12;14(6):1382. doi: 10.3390/ma14061382 (PMC8000002; doi:10.3390/ma14061382)
Supplement: Supplementary file 1 [file materials-14-01382-s001.pdf]

materials

Supplementary Material

Encapsulation and Enhanced Release of Resveratrol from Mesoporous Silica Nanoparticles for Melanoma Therapy

Diogo Marinheiro [1], Bárbara J. M. L. Ferreira [1,*], Párástu Oskoei [2], Helena Oliveira [2] and Ana L. Daniel-da-Silva [1,*]

1 Department of Chemistry & CICECO-Aveiro Institute of Materials, University of Aveiro, 3810-193 Aveiro, Portugal; diogomarinheiro@ua.pt (D.M.); barbaraferreira@ua.pt (B.J.M.L.F.); ana.luisa@ua.pt (A.L.D.-d.-S.)

2 Department of Biology & CESAM-Centre for Environmental and Marine Studies, University of Aveiro, 3810-193 Aveiro, Portugal; parastu.oskoei@ua.pt (P.O.); holiveira@ua.pt (H.O.)

A. UV-Vis calibration curves for RES quantification

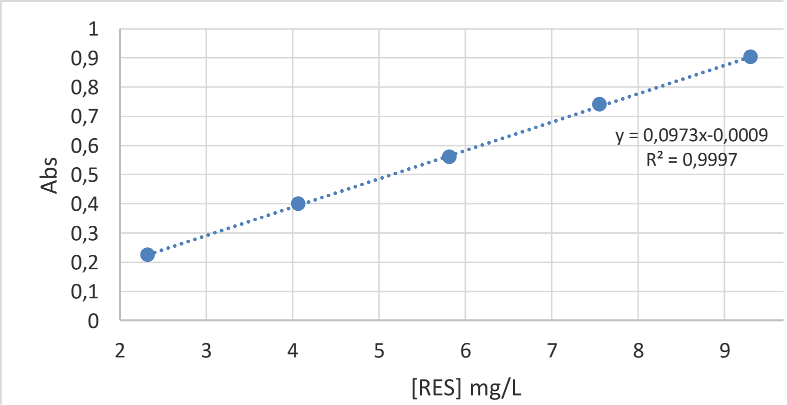

Figure S1. Calibration curve for RES quantification in PBS pH 7.4.

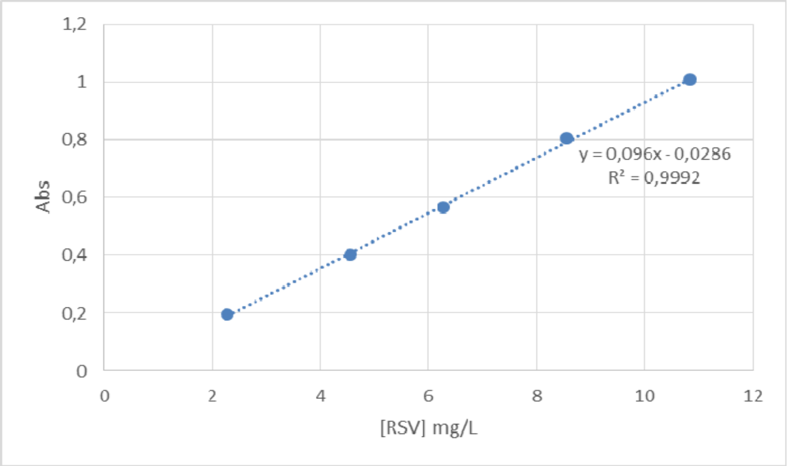

Figure S2. Calibration curve for RES quantification in PBS pH 5.2.

B. Resveratrol stability to isomerization during release studies

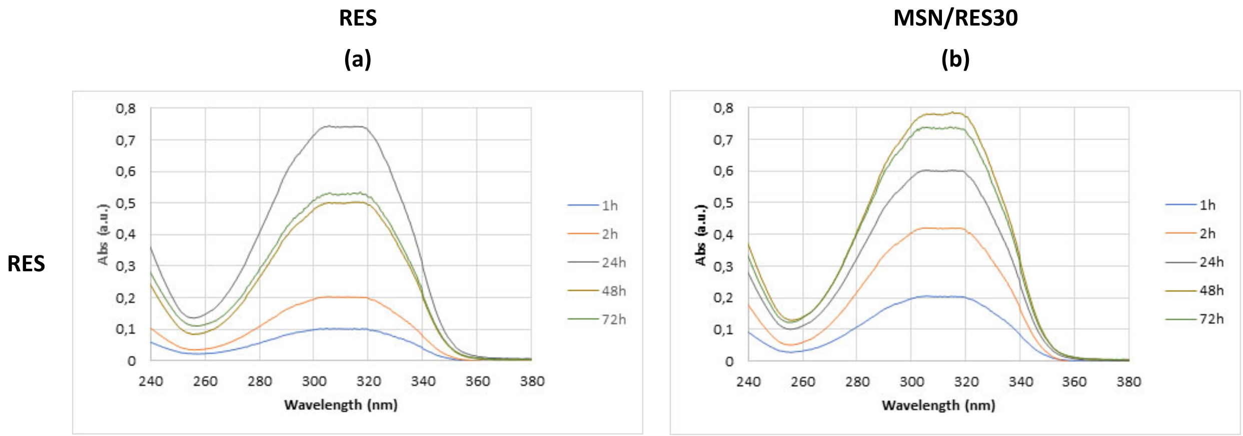

Figure S3. UV-Vis spectra (240-380 nm) of aliquots extracted along time during RES release studies at pH 5.2 of the (a) non-encapsulated RES and (b) loaded sample MSN/RES30.

C. Comparison of immersion and evaporation loading methods

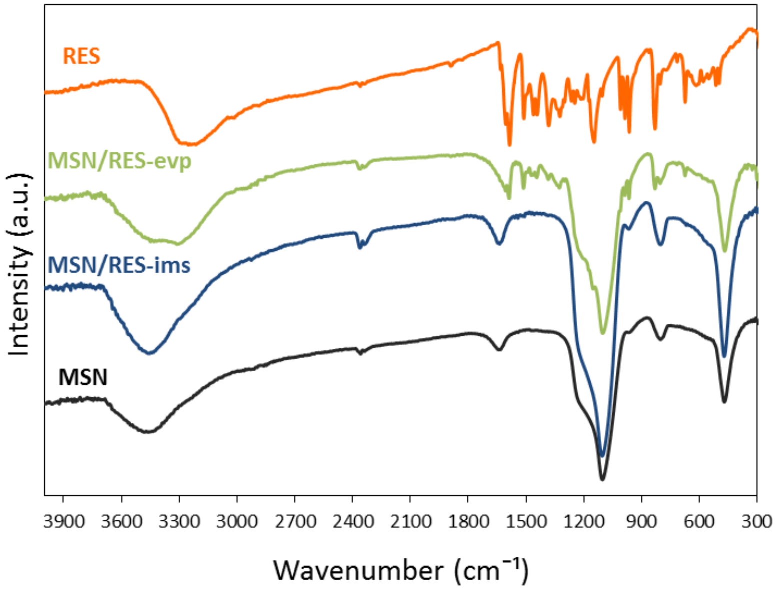

Figure S4. FTIR spectra of pristine MSNs, RES and loaded MSNs using the immersion (MSN/RES-ims) and the evaporation (MSN/RES-evp) methods. The amount of MSNs (100 mg) and RES (40 mg) available for loading was identical in both methods.

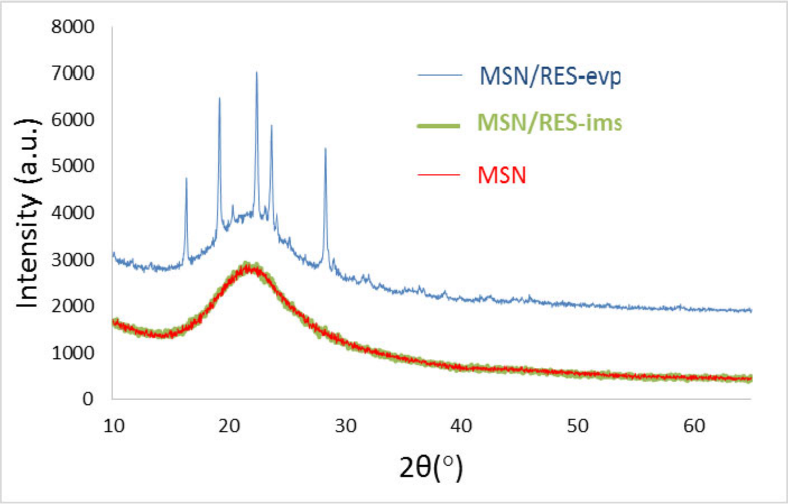

Figure S5. Wide angle XRD of pristine MSNs and loaded MSNs using the immersion (MSN/RES-ims) and the evaporation (MSN/RES-evp) methods.

D. Characterization of RES loaded MSNs

Table S1. Elemental analysis of MSNs and loaded samples MSN/RES30 and MSN/RES40

	**MSNs**	**MSN/RES30**	**MSN/RES40**
**%C**	0.040	20.21	32.47
**%H**	1.424	2.405	2.915

Table S2. Melting temperature and cristallinity degree of the samples.

	**Tpeak ºC**	**Crystalinity (%)**
**RES**	264.9	-
**MSN/RES30**	226.7	15.3
**MSN/RES40**	242.5	19.4
